# An easy and sensitive assay for acetohydroxyacid synthases based on the simultaneous detection of substrates and products in a single step

**DOI:** 10.1007/s00216-024-05613-1

**Published:** 2024-10-23

**Authors:** Annika Engelhardt, Marco Ebeling, Elisabeth Kaltenegger, Dorothee Langel, Dietrich Ober

**Affiliations:** Botanical Institute and Botanic Gardens, Kiel University, D-24098 Kiel, Germany

**Keywords:** Acetolactate synthase, Branched-chain amino acid biosynthesis, Derivatization, Ethyl chloroformate, Kinetics, Substrate specificity

## Abstract

Acetohydroxyacid synthase (AHAS, EC 2.2.1.6) catalyzes the first step in the synthesis of the branched-chain amino acids valine, leucine, and isoleucine, pathways being present in plants and microorganisms, but not in animals. Thus, AHAS is an important target for numerous herbicides and, more recently, for the development of antimicrobial agents. The need to develop new and optimized herbicides and pharmaceuticals requires a detailed understanding of the biochemistry of AHAS. AHAS transfers an activated two-carbon moiety derived from pyruvate to either pyruvate or 2-oxobutyrate as acceptor substrates, forming 2-acetolactate or 2-acetohydroxy-2-butyrate, respectively. Various methods have been described in the literature to biochemically characterize AHAS with respect to substrate preferences, substrate specificity, or kinetic parameters. However, the simultaneous detection and quantification of substrates and unstable products of the AHAS-catalyzed reaction have always been a challenge. Using AHAS isoform II from *Escherichia coli*, we have developed a sensitive assay for AHAS-catalyzed reactions that uses derivatization with ethyl chloroformate to stabilize and volatilize all reactants in the aqueous solution and detect them by gas chromatography coupled to flame ionization detection or mass spectrometry. This assay allows us to characterize the product formation in reactions in single and dual substrate reactions and the substrate specificity of AHAS, and to reinterpret previous biochemical observations. This assay is not limited to the AHAS-catalyzed reactions, but should be applicable to studies of many metabolic pathways.

**Graphical Abstract:**

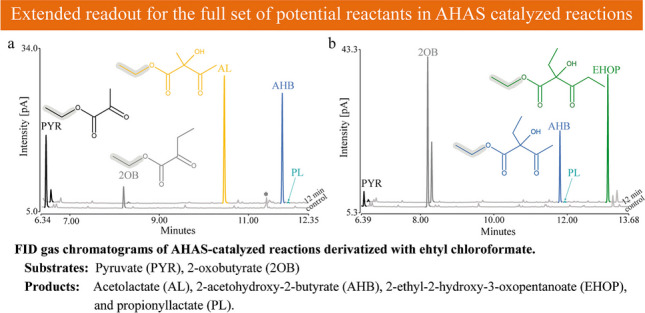

**Supplementary Information:**

The online version contains supplementary material available at 10.1007/s00216-024-05613-1.

## Introduction

Acetohydroxyacid synthase (AHAS, formerly called “acetolactate synthase,” EC 2.2.1.6) catalyzes the first step in the biosynthesis of branched-chain amino acids (BCAAs) in bacteria, plastids of plant cells, and mitochondria of fungi [1, 2]. Animals do not express this pathway making BCAAs essential for their diet [3]. AHAS is of substantial importance as a target for herbicides of various classes [3–8], such as sulfonylurea derivatives or imidalozes [9–13]. Inhibitors that are designed to target AHAS and that are considered harmless to vertebrates and other animals are being investigated as promising drugs for the treatment of pathogenic microbial [14–16] and fungal infections [13, 17, 18].

**Fig. 1:**
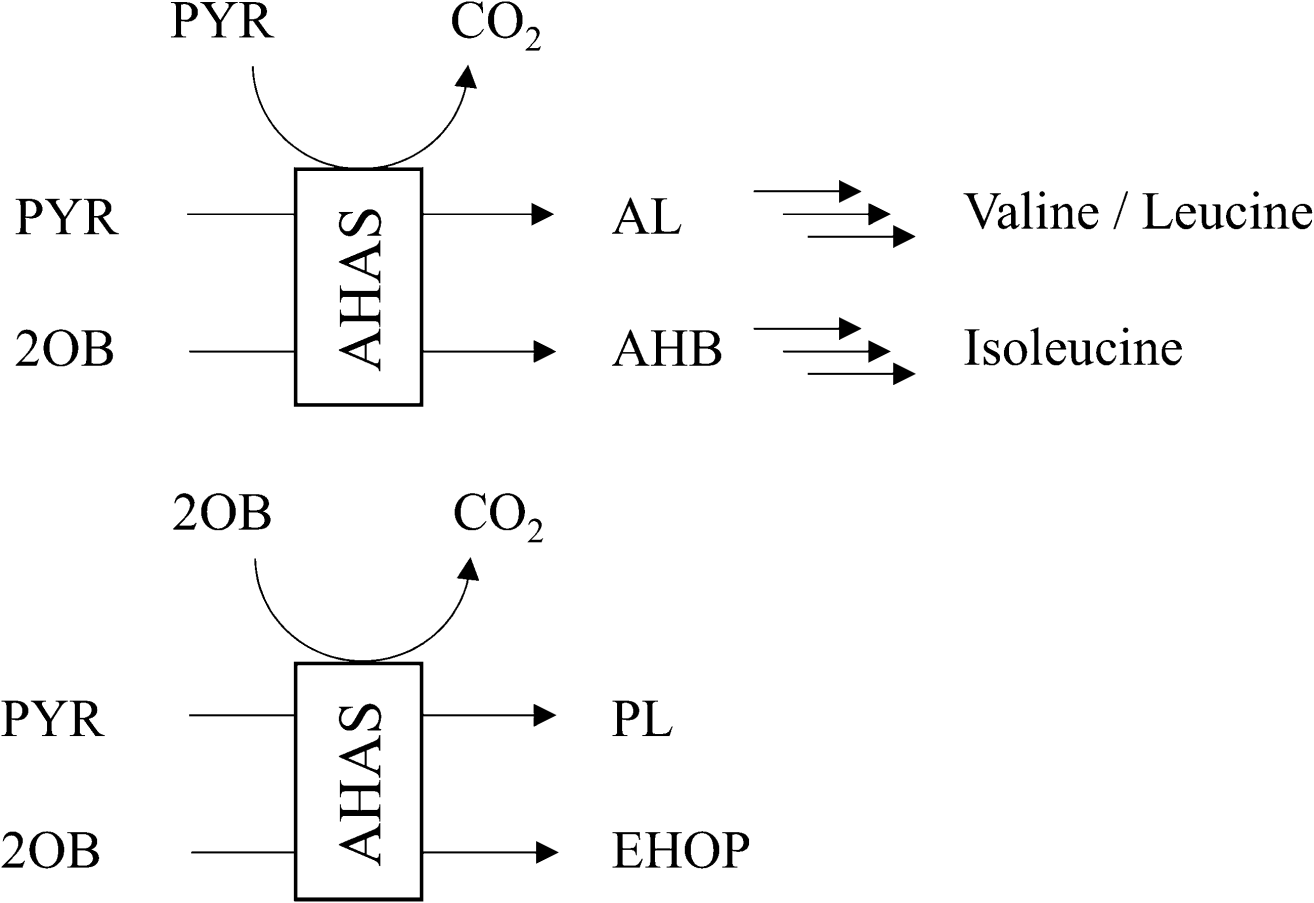
Reactions catalyzed by AHAS as established for branched-chain amino acid biosynthesis (top) and as predicted from previous studies (bottom). AHAS transfers the C_2_- or C_3_-moiety resulting from the activation of PYR or 2OB, respectively, to the various acceptor substrates. PYR, pyruvate; 2OB, 2-oxobutyrate; AL, 2-acetolactate; AHB, 2-acetohydroxy-2-butyrate; PL, 2-propionyllactate; EHOP, 2-ethyl-2-hydroxy-3-oxopentanoate

Due to the importance of AHAS in agriculture and medicine, the biochemical properties of this enzyme have been intensively studied and summarized in several reviews [2, 19, 20]. AHAS is a multimeric protein consisting of eight catalytic subunits (CSUs) and up to eight regulatory subunits (RSUs) [1, 3, 21–23]. The RSUs form the core of the complex to which the CSUs are attached. The CSUs are active as a dimer and in this form possess the entire catalytic machinery of AHAS [21, 24]. The RSUs regulate the AHAS by feedback inhibition in response to intracellular BCAA levels, particularly that of valine [19, 25]. AHAS depends on thiamine diphosphate to activate the first substrate under decarboxylation. In the case of pyruvate (PYR) as the activator substrate, hydroxyethyl thiamine diphosphate (HeThDP) is formed, of which the activated C_2_-moiety is transferred to a second 2-ketoacid as the acceptor substrate. If PYR is also the acceptor substrate for HeThDP, 2-acetolactate (AL) is formed as an intermediate in the biosynthesis of L-leucine and L-valine; if 2-oxobutyrate (2OB) is the acceptor substrate, 2-acetohydroxy-2-butyrate (AHB) is formed as an intermediate in the biosynthesis of L-isoleucine [26, 27] (Fig. 1). A comprehensive characterization of AHAS activity including the kinetics of the dual substrate reactions or substrate specificity has been hampered by the unavailability of appropriate assays. Distinguishing between the different products formed and the limited stability of these products, which tend to degrade by decarboxylation, has always been a challenge, requiring several post-incubation steps to detect at least some of the reaction partners [28–30].

Using the AHAS isoenzyme II of *Escherichia coli* (*Eco*AHASII) for which kinetic data is available in the literature that we used for comparative purposes [31–34], we have developed an easy, fast, and flexible assay for AHAS activity. This assay is based on the derivatization of all reactants directly in the aqueous reaction solution in a single step with ethyl chloroformate (ECF) followed by gas chromatography (GC) coupled with flame ionization detection (FID) or mass spectrometry (MS). This assay allows the sensitive detection, quantification, and, in the case of MS, also the identification of all substrates and products. Since standards of the reaction products are not commercially available, we have estimated the amount of product according to a calibration obtained by complete conversion of specific amounts of substrate. We used this assay to analyze the AHAS-catalyzed reaction with one and two different substrates and were able to determine the kinetic parameters even of the dual substrate reaction. The ability to analyze the amounts of substrates and products in parallel of the same enzymatic reaction allowed us to evaluate and reinterpret the different rates of product formation in dual substrate reactions. Analysis of substrate specificity revealed the inability of *Eco*AHASII to convert 2-ketoacids with branched side chains that compete with PYR for binding to the binding site of the acceptor substrate. These observations allowed the reinterpretation of previous observations with *Eco*AHASII [31–33, 35–37] and demonstrated the potential of the assay for further enzymatic analyses including, but not limited to, the further steps of BCAA biosynthesis.

## Material and methods

### Heterologous expression of *Eco*AHASII

The plasmid pET-GM [32] was obtained from Dr. Ronald Duggleby (University of Queensland, Brisbane, Australia). This construct encodes the *Eco*AHASII consisting of the CSU (59 kDa) with a 50-residue (5.43 kDa) *N*-terminal fusion encoding an oligohistidine tag, and the RSU (9.5 kDa) in its native form. Expression and purification were performed as described previously [32] and resulted in the co-purification of the RSU most likely due to complex formation (Fig. S1). All steps of protein purification in this study were protected from light as it has been reported that AHAS may be light sensitive [38]. Briefly, after transformation of the plasmid pET-GM into *E. coli* BL21(DE3), cultures were induced at OD_600_ of 0.6 to 0.8 with 0.5 mM IPTG and cultured for 20 h at 22°C on a rotary shaker. Harvested cells were resuspended in 10 ml lysis buffer (50 mM NaH_2_PO_4_xH_2_O, 300 mM NaCl, 5 mM β-mercaptoethanaol, 20 mM imidazole, pH 7.5) and sonicated twice for 7 min using a Branson sonifier (Heinemann, Schwäbisch Gmünd, Germany, cycle 40, duty 4). The supernatant was added to 200 µl of nickel-nitrilotriacetic agarose (Qiagen, Hilden, Germany) equilibrated with lysis buffer and incubated end-over-end at 4°C for 1 h. Purification and elution of recombinant AHAS were performed according to the manufacturer’s protocol with wash buffer (50 mM NaH_2_PO_4_, 600 mM NaCl, 5 mM β-mercaptoethanaol, 60 mM imidazole, pH 8.0) and elution buffer (50 mM NaH_2_PO_4_, 300 mM NaCl, 5 mM β-mercaptoethanaol, 270 mM imidazole, pH 8.0). Affinity-purified protein was rebuffered with assay buffer (0.05 M K_2_HPO_4_, 1 mM MgCl_2_, 1 mM thiamine pyrophosphate, 20 μM FAD, 1 μM DTT, pH 7.9) through a PD10 column (GE Healthcare, Chicago, IL, USA) and stored at − 80°C after the addition of 1% glycerol.

### AHAS activity assay based on indirect colorimetric detection of AL

The colorimetric assay of AHAS was first described by Westerfeld [39] and was used in our study with PYR as substrate according to an optimized protocol [40] for non-quantitative activity assays of enzyme preparations. The produced AL forms a color complex with creatine and naphthol after decarboxylation to acetoin. For our analyses, 10 µg of affinity-purified enzyme was incubated in 250 µl assay buffer containing 10 mM PYR at 30°C for 30 min. The reaction was stopped by addition of 25 µl of 6N H_2_SO_4_ and incubated for 15 min at 60°C to allow the formation of the corresponding diketones. Color complex formation was initiated by adding 275 µl of 5% (w/v) 1-naphthol solution in 4 M NaOH and 275 µl of 0.05% (w/v) creatine solution in H_2_O and incubating for 15 min at 60°C. Enzyme activity was indicated by a color change from brownish to pink.

### AHAS activity assay based on alkylation of substrates and products

For activity assays, affinity-purified enzyme at a concentration of 1–100 µg/ml was incubated at 30°C in a total volume of 200 µl assay buffer with varying concentrations of substrates. All substrates were prepared as 0.2 M stock solutions in assay buffer to ensure identical concentrations of all cofactors in the reactions. The substrates we used in this study were sodium pyruvate (PYR, CAS 113–24-6, Carl Roth, Germany; ACROS Organics, NJ, USA; Merck, Darmstadt, Germany), sodium 2-oxobutyrate (2OB, CAS 2013–26-5, Merck), 2-oxovaleric acid (2OV, CAS 1821–02-9, Merck), sodium 2-oxoisovalerate (2OIV, CAS 3715–29-5, ACROS Organics), sodium 3-methyl-2-oxopentanoate (3MOP, CAS 3715–31-9, Merck), sodium 4-methyl-2-oxopentanoate (4MOP, CAS 4502–00-5, Merck), and sodium 2-oxooctanoate (2OO, CAS 328–51-8, Fisher Scientific, Hampton, VA, USA). Reactions were stopped by adding 40 µl of 5% (w/v) NaOH. As recommended by Smart et al. [41] for sample preparations involving several steps prior to sample injection into the GC, we used 1 mM 2OV, 2OB, or 2OIV as an internal standard to compensate for possible metabolite losses and matrix effects and to allow accurate quantification. Substrates and products were derivatized with ECF (Sigma-Aldrich) to allow separation, detection, and quantification according to a protocol for methylation of ketoacids [41] in 2-ml safe-seal reaction tubes (Sarstedt, Nümbrecht, Germany). After 200 µl ethanol (donor of the ethyl group) and 50 µl pyridine (catalyst of the reaction), 50 µl ECF was added in two portions of 25 µl to the reaction mixture (ECF is toxic, use a fume hood!). Following the addition of each aliquot of ECF, the reaction was mixed intensively for 30 s with breaks after intervals of 10 s to open the tube to avoid overpressure due to gas formation [41, 42]. If the reaction contained high concentrations of substrates and products, an aliquot of the reaction was taken and diluted in such a way that the total concentration of substrates and products was less than 7 mM. The ethylated compounds were extracted into an organic phase by adding 400 µl of chloroform and stirring vigorously for 10 s. After adding 400 µl of NaHCO_3_ and stirring vigorously for 10 s, the upper, aqueous phase and the interphase containing any turbid matter were removed from the organic phase. To dry the organic phase, 100 mg Na_2_SO_4_ was added and centrifuged (12,000 × g for 30 s) after intense mixing for 10 s. The sample was transferred to a glass vial with a 300-µl insert (Phenomenex, Torrance, CA, USA) for GC analysis without any crystals that may be present at the bottom of the tube.

**Fig. 2:**
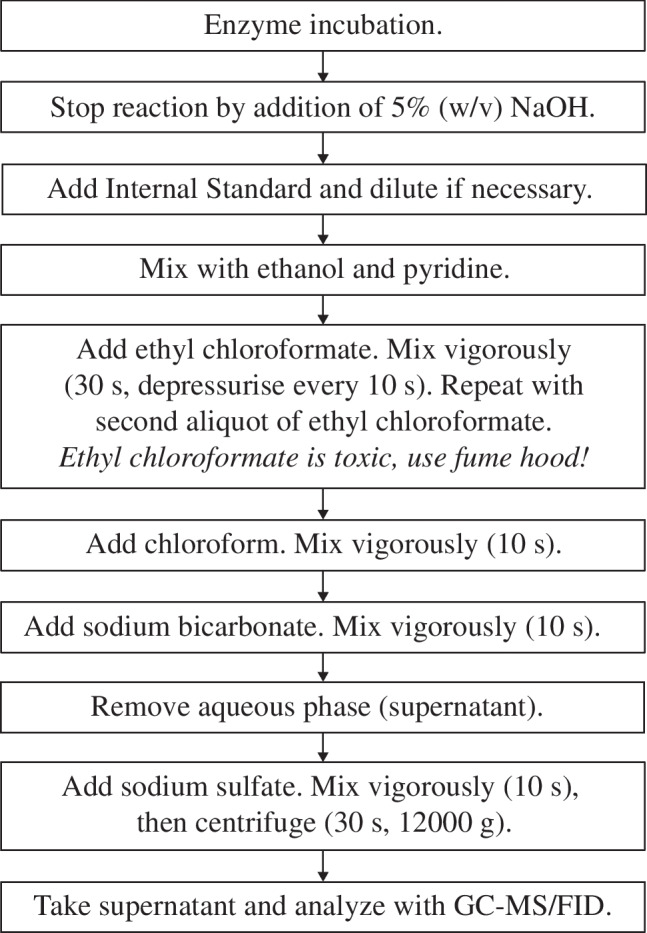
Flowchart of the experimental steps

GC data were obtained using a Thermo TSQ TRACE1300/1310 coupled to a Triple Quadrupole Mass Spectrometer (Thermo Fisher Scientific) equipped with a TG-5SILMS column (30 m × 0.25 mm i.d., 0.25 μm film thickness, Thermo Fisher Scientific). GC conditions were as follows: temperature program 50°C for 3 min, 50–200°C at 10°C/min, 200 to 280°C at 25°C/min followed by 1 min at 280°C. For FID, the samples were injected using a programmed temperature vaporization injector with the following GC parameters: split flow 10 ml/min, split ratio 1:10, purge flow 3 ml/min. The FID parameters were as follows: detection rate 10 Hz, 300°C constant temperature, air flow 350 ml/min, nitrogen flow 40 ml/min, hydrogen flow 35 ml/min. For MS detection, a split/splitless (SSL) injector was used with 1 ml/min helium as carrier gas, split ratio 1:10, MS transfer line 300°C. EI‑mass spectra were recorded at 70 eV (ion source temperature 280°C). Linear retention indices (RI) were calculated according to Kováts [43] with a reference set of a co-injected analytical hydrocarbon standard (C_10_-C_40_, Merck). Analytes were identified using MS spectra (Fig. S2c and Table S1) and RI in comparison with data from the NIST database, literature [41, 42], and a commercially available standard (ethyl 2OB, CAS 15933–07-0, AmBeed, Arlington Heights, IL, USA). The experimental steps for this method are summarized in the flowchart in Fig. 2.

## Results

### Chromatographic separation

**Fig. 3:**
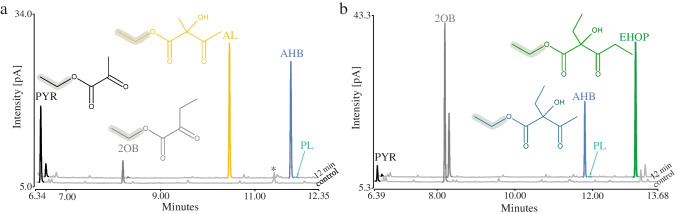
Representative FID gas chromatograms for the analysis of ECF-derivatized keto- and acetohydroxyacids. Reactions with two different ratios of substrates were set up, one with 5 mM pyruvate (PYR) and 1 mM 2-oxobutyrate (2OB) (**a**) and one with 1 mM PYR and 5 mM 2OB (**b**). Chromatograms show a standard reaction assay without enzyme (control) and after incubation for 12 min at 30 °C with 10 µg of affinity-purified *Eco*AHASII. Detectable products are 2-acetolactate (AL), 2-acetohydroxy-2-butyrate (AHB), 2-ethyl-2-hydroxy-3-oxopentanoate (EHOP), and propionyllactate (PL). The asterisk in **a** indicates traces of 2,2-dihydroxypropionate as a contaminant of PYR, which was shown to be converted by *Eco*AHASII (Fig. S2a)

Previous studies of AHAS have shown that the formation of the different products in reactions with more than one substrate depends on the ratio of the substrates [31, 44]. Therefore, a comprehensive biochemical characterization of AHAS enzymes requires the simultaneous detection of all substrates and products. We derivatized the ketoacid substrates and the acetohydroxyacid products with ECF to improve their volatility and, in the case of the products, their stability, since the acetohydroxyacids tend to degrade easily by oxidative decarboxylation to their respective diketones [1, 45]. Hušek [46] has already emphasized that the pyridine-catalyzed chloroformate-mediated ester formation proceeds smoothly at the analytical microscale. ECF derivatives have been described to be easily detectable at high resolution by GC [47]. For our analysis, we used GC coupled to a FID and a mass spectrometer (MS). Both detectors were coupled to identical GC columns in the same GC, allowing the described assay to be optimized in parallel for both detection methods (for MS data, total ion chromatograms (TICs) were used for quantification, Supplemental information). To allow the formation of all possible products, two reactions with different ratios of the two substrates PYR and 2OB were set up with 10 µg of affinity-purified *Eco*AHASII and stopped after 12 min before the substrates were completely converted and subsequently derivatized with ECF and detected by FID (Fig. 3) and MS (Fig. S2). Figure 3a shows the FID chromatogram of the reaction containing 5 mM PYR and 1 mM 2OB. In the negative control without enzyme addition, two distinct peaks were detected and identified as ethylated PYR and 2OB (mass spectra in Fig. S2c). In the reaction with enzyme, two distinct peaks were detected that were not present in the negative control. These were identified as ethyl esters of AL and AHB. Figure 3b shows the FID chromatogram of the second reaction with 1 mM PYR and 5 mM 2OB, a ratio that favored the formation of 2-ethyl-2-hydroxy-3-oxopentanoate (EHOP), a product formed from two molecules of 2OB without PYR as substrate. In both reactions’ traces of 2-propionyllactate (PL), the product formed from 2OB as activator substrate and PYR as acceptor substrate could be detected, a reaction previously described from *E. coli* and *Bacillus subtilis* [30, 37]. Overall, we could show that the peaks are well separated, allowing easy integration of the peak areas in both the FID and the MS chromatograms (Fig. S2a and S2b). The performance and sensitivity of this method were demonstrated by the detection and quantification of 2,2-dihydroxypropionate as an impurity (Fig. 3a, Fig. S2a and S2c) in three tested batches of purchased PYR (supplier guaranteed a purity greater than 99%). The product resulting from the conversion of 2,2-dihydroxypropionate by AHAS (Fig. S2a and S2c) was also well detected, allowing such unexpected conversions to be considered in the interpretation of the data obtained. All RI values and the most prominent peaks of the MS spectra are summarized in Table S1. Note that the ECF derivatives of all reaction components eluted after the front peaks caused by the solvent and derivatization chemicals. This observation was the argument for using ECF instead of methyl chloroformate (MCF), although almost all of the experiments presented here were initially performed with MCF (Table S2), as MCF derivatization is reported to be the best in terms of derivatization yield and reproducibility [47]. In the case of MCF, the peak caused by the pyridine used as a derivatization catalyst eluted after the peak of the MCF derivative of PYR and before the derivatives of the other reaction components, thus interfering with the simultaneous integration of the peaks resulting from all reactants of the AHAS-catalyzed reaction.

### Method validation

**Fig. 4:**
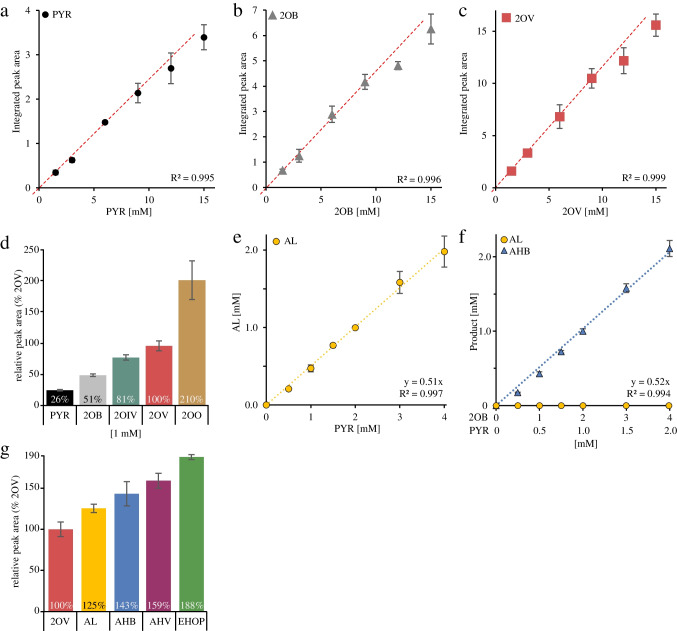
FID detection of assay components, ethylated with ECF. Correlation between the integrated peak areas of the ECF derivatives of the substrates and the substrate concentration used for derivatization [1–15 mM] for pyruvate (PYR, **a**), 2-oxobutyrate (2OB, **b**), and 2-oxovalerate (2OV, **c**). The red dashed trend line crosses the origin and represents only the values for substrate concentrations from 0 to 9 mM. **d** FID response factors of the different ketoacids. PYR, 2OB, 2-oxoisovalerate (2OIV), 2OV, and 2-oxooctonate (2OO) were mixed (1 mM each) and derivatized with ECF. The integrated peak areas were related to that of 2OV (set at 100%). **e** Correlation between the amount of PYR as the sole substrate and the peak area of the product 2-acetolactate (AL). **f** Correlation between the concentrations of PYR and 2OB (in a ratio of 1:2) and the peak area of the product 2-acetohydroxy-2-butyrate (AHB). **g** FID response factors of the products (1 mM each) AL, AHB, 2-acetohydroxy-2-valerate (AHV), and 2-ethyl-2-hydroxy-3-oxopentanoate (EHOP) obtained from the values of the complete substrate conversions shown in **e** and **f** and from the incubation of 1 mM PYR in the presence of 5 mM 2OV resulting in the formation of AHB and the incubation of 2 mM and 4 mM 2OB as sole substrate for the formation of EHOP. 2OV was used as a standard and was set to 100%. Each value represents the mean ± SE of triplicate determinations

Linearity for substrate derivatization was tested by derivatizing the substrates PYR, 2OB, and the substrate homolog 2OV, which was used as an internal standard in our study, at concentrations ranging from 1 mM up to 15 mM in the standard reaction. Quantification of the resulting peak areas confirmed linearity for concentrations up to 9 mM (Fig. 4a–c, Fig. S3a–c). Therefore, enzyme reactions set up with higher concentrations of ketoacids were diluted prior to derivatization so that the total concentration of all substrates, products, and internal standard was less than 7 mM. Of note, comparison of the peak area resulting from the derivatization of different amounts of 2OB with that of equal amounts of purchased ethyl-2OB showed a derivatization efficiency of approximately 26% for 2OB concentrations up to 9 mM and approximately 24% for 12 mM. The data in Fig. 4a–c (and Fig. S3a–c) suggest different response factors for the three ketoacids tested. Therefore, we compared the response factors by analyzing a mixture of equal amounts of different ketoacids. Figure 4d (and Fig. S3d) shows that the response factors related to that of the internal standard 2OV depend on the length and the branching pattern of the ketoacids. Note that in our case the response factors are not only the functions of the detectors used, but also of the derivation efficiency of the components. The result was the same regardless of whether or not 5 µg/ml of inactivated enzyme protein was added to the substrate mixture.

AHAS reactions described in the literature often contain substrates in the millimolar range [1, 31, 33, 48]. Due to these large amounts of substrates, the quantification of product formation per time should be more accurate for quantifying enzyme activity than quantification of substrate consumption, especially at low enzyme activities. However, due to the instability of the products of typical AHAS reactions, standards of the products are not available. Therefore, direct quantification by comparison with a calibration curve of the corresponding product standard was not possible.

To estimate the amount of products, we took advantage of the observation that AHAS converts substrates almost completely to their respective products. Therefore, we set up enzyme reactions (50 µg/ml) with defined concentrations of the substrates and extended incubation times to allow complete conversion of at least one of the substrates. 2OV served as an internal standard at a concentration of 1 mM. Using PYR as the only substrate, we defined that the peak area of the resulting product AL represents the half of the amount of the substrate at the beginning of the reaction (Fig. 4e and Fig. S3e). The same was done with PYR and 2OB in a ratio of 1:2 as we have observed that this ratio of substrates leads to the formation of AHB without the formation of AL. Figure 4f shows that AL is indeed not produced. Therefore, we defined the peak area of the product to represent the same amount as the substrate PYR at the beginning of the reaction. Note that we also detected the formation of PL in this reaction with a peak area of approximately 7.5% of that of AHB, which opens up the possibility of including this conversion in further studies. However, for our project, we did not include this product in further analysis. Figure 4e and f (and Fig. S3e and S3f) confirm the linear correlation between the amount of substrate and the relative peak areas of the products with respect to the internal standard. For the product 2-acetohydroxy-2-valerate (AHV), we have defined that after complete conversion of 1 mM PYR in the presence of 5 mM 2OV, the peak area represents 1 mM AHV. As with the formation of AHB, this substrate ratio prevents the formation of AL. For EHOP, which is formed from two molecules of 2OB, we set up two reactions with 2 mM and 4 mM 2OB. Since we did not have complete conversion, we defined the peak area of EHOP to represent half of the amount of 2OB converted. Figure 4g (and Fig. S3g) shows a direct comparison of the response factors of the products AL, AHB, AHV, and EHOP (in % relative to 2OV as internal standard). This approximation of product quantification allowed us to test the performance of this assay for AHAS activity and to compare the data obtained to that of the literature using the AHAS II of *Escherichia coli*.

Based on our experience with method validation, we would like to make the following recommendations: Solutions of substrates and internal standards should be prepared fresh every few days. When several samples were derivatized in parallel, substrates and products were stable for several hours at room temperature after stopping the reaction with NaOH. As already described by Madsen et al. [42], the derivatized samples were stable at room temperature for several days and could easily be stored at − 20°C for several days until further analysis.

### Kinetics of *Eco*AHASII

**Fig. 5:**
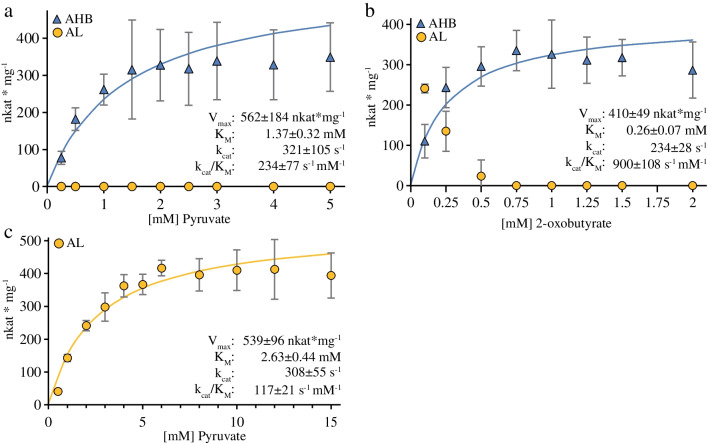
Michaelis–Menten plots for affinity-purified *Eco*AHASII. *Eco*AHASII was incubated in a standard reaction assay with varying amounts of substrate for 8 min. **a** Formation of 2-acetohydroxy-2-butyrate (AHB) as a function of the pyruvate (PYR) concentration [0.25–5 mM] in the presence of 5 mM 2-oxobutyrate (2OB) with 2.5 µg/ml *Eco*AHASII. The *V*_max_ and the *K*_M_ values were calculated to be 562 ± 184 nkat/mg and 1.37 ± 0.32 mM, respectively. **b** Formation of AHB as a function of the 2OB concentration [0.1–2 mM] in the presence of 10 mM PYR with 1 µg/ml *Eco*AHASII. The *V*_max_ and the *K*_M_ values were calculated to be 410 ± 49 nkat/mg and 0.26 ± 0.07 mM, respectively. **c** Formation of 2-acetolactate (AL) as a function of the PYR concentration [1–15 mM] with 5 µg/ml *Eco*AHASII. The *V*_max_ and the *K*_M_ values were calculated to be 539 ± 96 nkat/mg and 2.63 ± 0.44 mM, respectively. Each point represents the mean ± SE of triplicate determinations. For *k*_cat_ calculation, we assumed the complete CSU/RSU hexadecameric complex to represent the molecule of active AHAS according to Lonhienne et al. [23]

To estimate the kinetic properties of *Eco*AHASII, we choose a compromise between incubation times as short as possible but long enough to obtain a sufficient amount of product for proper quantification and used standard incubation times of 8 min with 5 µg/ml of affinity-purified enzyme. Figure 5a and b show the kinetics of the dual substrate reaction with PYR and 2OB to produce AHB, a reaction for which there are almost no data in the literature. In Fig. 5a, the enzyme reactions contained 5 mM 2OB and concentrations of PYR ranging from 0.25 to 5.0 mM. A Lineweaver–Burk plot approximation resulted in a *K*_M_ value for PYR as substrate for the generation of the HeThDP in the binding site for the activator substrate of 1.37 mM. In Fig. 5b, the enzyme reactions contained 10 mM PYR and concentrations of 2OB ranging from 0.125 to 2.0 mM, resulting in a *K*_M_ value of 0.26 mM for 2OB as acceptor substrate. Notably, we observed the production of AL only at very low concentrations of 2OB, suggesting that the affinity of the binding site for the acceptor substrate is much higher for 2OB than for PYR (Fig. 5b). This interpretation is supported by the data in Fig. 5c, which shows the AL formation as a function of increasing concentrations of PYR as the sole substrate. A Lineweaver–Burk plot approximation resulted in a *K*_M_ value of 2.63 mM. We calculated the catalytic efficiency (*k*_cat_/*K*_M_) of *Eco*AHASII with PYR at the binding site for the acceptor substrate to be 117 s^−1^ mM^−1^, which is only 50% of the efficiency of the site for HeThDP formation (234 s^−1^ mM^−1^) and almost eight times less than for 2OB as acceptor substrate (900 s^−1^ mM^−1^). Previous analyses of the kinetic data (summarized in Table 1) fit well with our data and show the advantages of being able to analyze also the *K*_M_ values for the dual substrate reactions. The slightly decreasing values of *V*_max_ with increasing concentration of 2OB (Fig. 5b) could indicate an inhibition by this substrate, an observation that was briefly mentioned earlier [44].

**Table 1:** Kinetic data for *Eco*AHASII with pyruvate as sole substrate reported in the literature

*K*_M_ [mM]	*k*_cat_ s^−1^	Publication
2.6	66.7	[32]
10.6	-	[31]
4.76	-	[49]
5.0	-	[50]
5.2	23.3	[34]
6.6	40.3	[37]

### Preference for 2OB as acceptor molecule

**Fig. 6:**
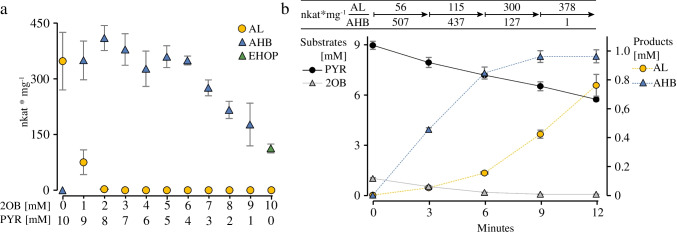
Competition assay with pyruvate (PYR) and 2-oxobutyrate (2OB) as substrates. **a** Enzyme activities for the formation of 2-acetolactate (AL), 2-acetohydroxy-2-butyrate (AHB), and 2-ethyl-2-hydroxy-3-oxopentanoate (EHOP) in reactions with different ratios of the two substrates PYR and 2OB (0–10 mM and 10–0 mM, respectively). Incubation for 8 min with affinity-purified *Eco*AHASII (5 µg/ml, 25 µg/ml in the reaction with 0 mM PYR and 10 mM 2OB). **b** Time course of substrate and product concentrations in a reaction with 9 mM PYR and 1 mM 2OB and 5 µg/ml affinity-purified *Eco*AHASII. A 200-µl aliquot was taken every 3 min, the reaction was stopped, and substrates and products were quantified. Substrate scale from 0 to 9 mM, product scale from 0 to 1 mM. Each point represents the mean ± SE of triplicate determinations

The observation that in the dual substrate reaction the catalytic efficiency of *Eco*AHASII for 2OB is higher than for PYR as an acceptor substrate has already been described [28, 31]. They found that equivalent rates of AHB and AL production with *Eco*AHASII are only observed when the concentration of PYR is approximately 180-fold higher than that of 2OB [31]. The data shown in Fig. 5b could suggest that at a substrate ratio of 10 mM PYR to 0.16 mM 2OB the two competing reactions occur with equal activity. However, our analyses show that in those reactions that started with 2OB concentrations lower than 0.75 mM, no 2OB was detectable after the 8-min incubation time, suggesting that AL is not produced simultaneously with AHB, but only after 2OB has been almost completely converted. This phenomenon was further analyzed in a substrate preference assay by setting up 11 reactions with different ratios of the two substrates, PYR and 2OB, ranging from 10 mM PYR and 0 mM 2OB to 0 mM PYR and 10 mM 2OB in one millimolar steps with 1 µg/ml *Eco*AHASII for 8 min (Fig. 6a). The amount of AL, which is the only product in the incubation with 10 mM PYR and 0 mM 2OB, decreases significantly with increasing amounts of 2OB and was already almost undetectable (< 10 µM) in the reaction with 8 mM PYR and 2 mM 2OB. In this reaction and in those with lower amounts of PYR, AHB is the only reaction product. The amount of AHB production decreases in those reactions with PYR concentrations less than 4 mM, most likely due to PYR limitation at the activator site where PYR is converted to HEThDP. Only in the reaction containing only 10 mM 2OB, EHOP, resulting from the condensation of two molecules of 2OB, could be detected as the exclusive product, an activity described for bacterial AHAS [51]. Note that no 2OB could be detected at the end of the 8-min incubation in the reaction started with 9 mM PYR and 1 mM 2OB, suggesting that AL production might only be possible only after almost complete conversion of 2OB and that the formation of the two different products is not constant during the reaction.

To analyze the amount of available substrates and the effect on AL production with time during the enzymatic reaction, we repeated the reaction with 9 mM PYR and 1 mM 2OB and analyzed reaction aliquots after 3, 6, 9, and 12 min. Figure 6b and Fig. S4 show that the specific activities for the 3-min intervals are not constant over the 12-min incubation period. Despite the low initial concentration of 2OB of only 1 mM, the AHB production rate is high (507 nkat/mg and 437 nkat/mg) during the first 6 min and decreases drastically to only 127 nkat/mg after 9 min, when almost all 2OB has been converted to AHB and to only 1.3 nkat/mg after 12 min (values refer to FID data). Only under these conditions with very low concentrations of 2OB (< 0.05 mM), which are completely different from the substrate ratio at the beginning of the reaction, PYR binds to the site for the acceptor substrate and is converted to AL. As a result, the specific activity increases from 56 nkat/mg and 115 nkat/mg after 3 and 6 min to 300 nkat/mg and 378 nkat/mg after 9 and 12 min of incubation. Our data show that the two reactions forming AL and AHB essentially occur sequentially and not simultaneously.

### Substrate specificity

**Fig. 7:**
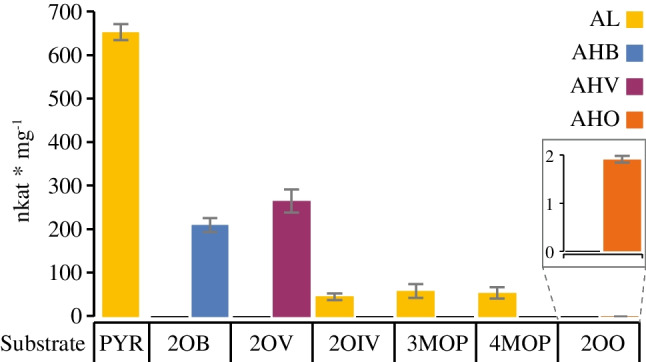
Specific activity of *Eco*AHASII with different ketoacids. *Eco*AHASII was incubated with either 6 mM PYR when given as sole substrate or with 1 mM PYR as donor substrate and 5 mM acceptor substrate. Apart from the reaction with PYR as sole substrate, which leads to the formation of AL, two products can be formed in the dual substrate reaction, depending on whether the acceptor substrate is PYR (AL formation) or one of the other substrates (leading to the formation of the products AHB, AHV, and AHO). 5 µg/ml affinity-purified *Eco*AHASII, except the incubation of PYR and 2OO (100 µg/ml). Quantification based on FID. Each bar represents the mean ± SE of triplicate determinations. 2OB, 2-oxobutyrate; 2OIV, 2-oxoisovalerate; 2OO, 2-oxoctonate; 2OV, 2-oxovalerate; AHB, 2-acetohydroxy-2-butyrate; AHO, 2-acetohydroxy-2-octonate; AHV, 2-acetohydroxy-2-valerate; AL, acetolactate; PYR, pyruvate

In order to test the usability of this assay with substrates that are not the standard substrates, we have analyzed the substrate preferences of *Eco*AHASII. We tested, in addition to PYR and 2OB, five other ketoacids as substrates that differ in the length and the branching of the side chains: 2-oxovalerate (2OV), 2-oxoisovalerate (2OIV), 3-methyl-2-oxopentanoate (3MOP), 4-methyl-2-oxopentanoate (4MOP), and 2-oxooctonate (2OO). PYR concentrations were 6 mM in the single substrate reaction and 1 mM in the two substrate reactions with one of the other substrates at a concentration of 5 mM. Figure 7 shows that *Eco*AHASII was only able to convert those substrates with a linear side chain, i.e., in addition to PYR in the single substrate reaction (653 nkat/mg), also 2OB (210 nkat/mg), 2OV (265 nkat/mg, product is 2-acetoyhdroxy-2-valerate, AHV), and, at a very low rate, 2OO (2 nkat/mg, product is 2-acetohydroxy-2-octonate). In all these reactions, no AL is formed, indicating that under these reaction conditions PYR is no acceptor substrate. *Eco*AHASII does not use molecules with a branched side chain as an acceptor substrate for the activated C_2_-moiety. Instead, AL is formed, but with a specific activity that is lower than in the absence of the second substrates. While AL is formed with an activity of 142 nkat/mg in a reaction containing 1 mM PYR as sole substrate (see Fig. 5c), the activity is reduced to 45 nkat/mg, 59 nkat/mg, and 52 nkat/mg in the presence of 2OIV, 3MOP, and 4MOP, respectively. Of note, these compounds are the last intermediates in the biosynthetic sequence leading to valine, isoleucine, and leucine, respectively. Therefore, Daily and Cronan [52] and Barak et al. [31], who had already observed such an inhibition, suggested that by converting minute amounts of these substrates, AHAS shows reduced activity due to feedback inhibition by the corresponding amino acids. Our data confirm that PYR is bound as acceptor substrate competing with the second substrate, which is not converted.

## Discussion

Several assays have been described in the literature to characterize the enzymatic properties of AHAS. However, none of these assays is capable of simultaneously detecting and quantifying the substrates and products of a typical AHAS-catalyzed reaction, i.e., ketoacids and acetohydroxyacids, respectively. Such a simultaneous chromatographic method has been considered necessary for the accurate analysis of AHAS-catalyzed reactions by several authors [28, 29, 31, 45, 53]. Therefore, the aim of our work was to develop a versatile assay to overcome these limitations by simultaneously analyzing substrate and product amounts after derivatization to allow separation and detection by GC-FID/MS. The assay presented here allows the reproducible quantification of each individual substrate and product not only in reactions with PYR as the sole substrate, a reaction involved in the biosynthesis of valine and leucine, but also in the dual substrate reactions characteristic of the biosynthesis of isoleucine. In addition, this assay allows the characterization of the substrate specificity of AHAS and should be applicable also to other metabolic pathways.

### Selected assays for AHAS activity described in the literature

A well-established method for the determination of AHAS activity is the colorimetric detection of the acetohydroxyacids after acidic decarboxylation and reaction with creatine and α-naphthol. The colorimetric reaction was first described in 1945 by Westerfeld [39] with later adaptations [40, 53]. This assay is characterized by an excellent sensitivity and simplicity. However, this method does not allow discrimination between the different products in dual substrate reactions. This is also the limit of several assays for AHAS proposed later [53–56]. Gollop et al. [28] were the first to develop a method that allowed to distinguish between the different products of the AHAS-catalyzed reaction. The products are decarboxylated to their respective diketones and analyzed by GC. The disadvantage of this method is the laborious extraction of the volatile diketones by an “air distillation,” which does not allow the simultaneous detection of substrates and products and requires a lot of routines to obtain reproducible results. Recently, Hui et al. [29] developed an HPLC-based method to quantify substrates and products for their studies of the catabolic acetolactate synthase reaction. PYR and the decarboxylation products of AL were derivatized in two separate reactions, a method that was later adapted to study reactions with more than one substrate [45]. Dual substrate reactions were also studied using a direct HPLC/UV-based method. Because this assay does not stabilize the products by derivatization, its applicability is limited to specific research questions [30].

### Advantages of the AHAS assay based on ECF derivatization

The direct alkylation of the substrates and products with ECF allowed the development of an assay for AHAS that is simple, versatile, and sensitive. The main advantages are:

1. Substrates and products of the AHAS-catalyzed reaction are derivatized in a single reaction and are detected, quantified and, in the case of MS detection, identified with a single chromatographic method. Substrate turnover and product formation can be observed simultaneously at any number of time points during the reaction, allowing the study of all aspects of the enzyme reaction, including aspects such as substrate limitations, substrate or product inhibition, or different kinetic parameters in the dual substrate reactions. In addition, the proposed method allows a comprehensive characterization of the substrate specificity of AHAS. It allows the use of unusual ketoacids as substrates, since the derivatization targets the functional group independently of the side chain structure.
2. ECF derivatization has been shown to provide sufficient yield and reproducibility and is not limited to the “classical” substrates of the AHAS reaction. Thus, we benefit from the advantages of the chloroformate ester derivatization demonstrated by Hušek [46], such as the instantaneous reaction without the need to exclude water, negligible reagent costs, simplified sample preparation that eliminates the need to isolate analytes from a matrix, and the ability to simultaneously determine a wide range of carboxylic acids. ECF derivatives are easily separated by GC. As early as Gollop et al. [28] demanded that it should be important to be able to quantify the efficiency of the conversion of the reaction partners to the derivatives analyzed by GC. Here, by comparing equimolar amounts of an ethylated ketoacid obtained as commercially available standard with the ketoacid derivative resulting from ECF derivatization, we have been able to show that the derivatization efficiency is about 26% over a wide range of concentrations. For studies that do not require the detection and quantification of PYR, derivatization with MCF is also possible (data see Table S2). Also, for MCF the derivatization efficiency is described to be constant over a wide array of possible substrates [46].
3. The high sensitivity and reproducibility of the method allowed us to detect enzyme activities in the range of more than 1000 nkat/mg to 2 nkat/mg in this study and is limited only by the detectability of the respective product. This observation is in agreement with previous studies describing the high sensitivity of methods based on chloroformate ester detection [42]. We have shown that even small amounts of PL, the product resulting from the transfer of an activated C_3_-moiety to PYR, are clearly detectable. In addition, we were able to identify contaminations of substrate batches. In the case of PYR the impurity was converted by AHAS, an activity that could be separated from the conversions used for enzyme characterization.
4. By using the strategy of complete substrate conversion by *Eco*AHASII we have solved the problem resulting of unavailability of proper product standards (whether derivatized or not) to a degree that has been shown to be good enough to allow accurate quantification of product formation.
5. Flexibility in method application results from the use of different detectors, such as FID and MS in this study. Both detectors have been shown in this study to allow accurate quantification by using the peak areas of the FID signal or the TIC in the case of MS detection in relation to that of an internal standard. Therefore, one of these detectors is sufficient to benefit from most of the advantages of the presented assay. The different response factors for the individual substrates and products can be easily determined with both detection methods tested. MS also allows the interpretation of the structural properties of the products, especially in cases where substrates other than the standard substrates are used. Single ion chromatograms allow analysis for the presence of specific fragments at low levels.
6. Flexibility in method application also results from the type of column used for GC separation. Since the AHAS-catalyzed reaction generates a new stereocenter in the reaction product by the transfer of the activated acetaldehyde, the use of GC columns that separate enantiomers would allow studies of the stereochemistry of the AHAS-catalyzed reaction and of other reactants with stereocenters.

### New characteristics of *Eco*AHASII described based on the new assay

The presented assay allows the kinetic study of AHAS not only with respect to the single substrate reactions but also to the dual substrate reactions with any combination of substrates. The competition of the two acceptor substrates, PYR and 2OB, and the formation of AL and AHB, respectively, has been intensively studied in the past. Barak et al. [31] analyzed this substrate competition over a wide range of substrate concentrations and found that the ratio of product formation rates to the ratio of substrate concentrations is related by an enzyme constant, the *R*-value. However, the authors were unable to detect the drastic shifts in substrate concentration during the 6-min incubation period. Using the new ECF-based method, we were able to analyze the shifts in substrate consumption and product formation over time and are able to show that the formation of AL and AHB occurs in a temporal sequence rather than as simultaneous competitive reactions. Contrary to Barak et al. [31], our results show that the *R*-value is not an enzyme constant. In addition, the assay allows also the unambiguous detection of the compounds PL and EHOP as the products of *Eco*AHASII transferring an activated C_3_-moiety resulting from 2OB as activator substrate to PYR and 2OB, respectively.

Another quite unexplored aspect is the substrate specificity, which we have analyzed for *Eco*AHASII using some substrate homologs. The ability to detect the substrates and the products allows to distinguish between the different reactions of the dual substrate reactions. We were able to show that *Eco*AHASII accepts ketoacids with unbranched side chains as substrate. Ketoacids with branched side chains are not converted, including the intermediates of BCAA biosynthesis, i.e., 2OIV, 3MOP, and 4MOP as precursors for valine, isoleucine, and leucine, respectively. Instead, 2OIV, 3MOP, and 4MOP inhibit the conversion of two molecules of PYR to AL by competing with PYR at the binding site for the acceptor substrate and not, as discussed in the literature for 2OIV and 3MOP [44, 52], by a feedback mechanism due to the formation of small amounts of the corresponding amino acid.

### Possible applications of the assay

**Fig. 8:**
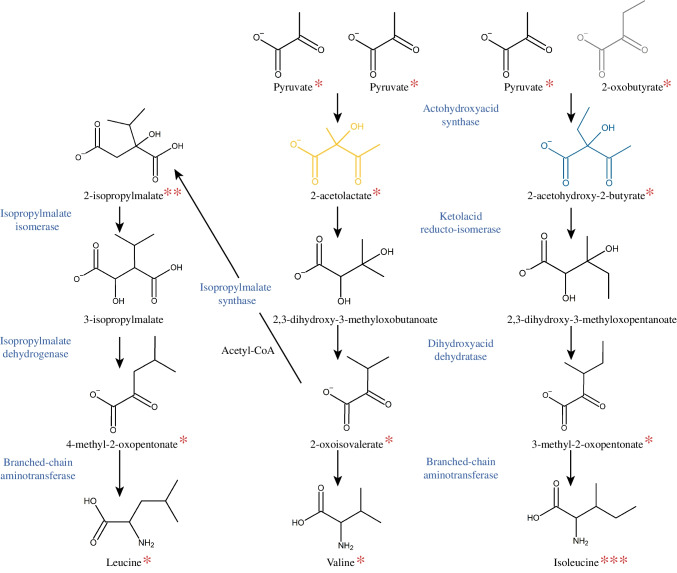
Key enzymes and metabolites of BCAA biosynthesis. 11 of the 14 metabolites were previously detected by GC as chloroformate esters. *Derivatized with ECF in this work (Table S1, for MCF Table S2); **Derivatized with MCF by Smart et al. [41]; ***Derivatized with ECF by Qiu et al. [57]

The flexibility of the AHAS assay presented, which allows the detection of a wide range of reaction compounds that are small organic acids or compounds containing amino groups after chloroformate-mediated ester formation, will encourage further studies of substrate specificity. Such studies will include testing for the ability of AHAS to convert compounds that are being tested as potential herbicides or antimicrobial agents using AHAS as a target. If conversion occurs, the reaction products must also be considered in a risk/benefit analysis. Since this assay is not only limited to the reaction of AHAS, many other reactions can be analyzed. These include the other reactions involved in BCAA biosynthesis. Of the 14 compounds involved in these pathways, 11 have been successfully detected as chloroformate esters in the literature or in this study. The detection of the further intermediates 3-isopropylmalate, 2,3-dihydroxy-3-methylbutyrate, and 2,3-dihydroxy-3-methylpentanate, is very likely (Fig. 8). Since there are many metabolic conversions involving small organic acids or compounds with amino groups, we believe that this assay can be easily adapted for other research questions.

## Supplementary Information

Below is the link to the electronic supplementary material.

Supplementary file1 (PDF 1.12 MB)

## Abbreviations

2OB: 2-Oxobutyrate
2OIV: 2-Oxoisovalerate
2OO: 2-Oxooctanoate
2OV: 2-Oxovalerate
3MOP: 3-Methyl-2-oxopentanoate
4MOP: 4-Methyl-2-oxopentanoate
*Eco*AHASII: AHAS isoenzyme II of *Escherichia coli*
AHAS: Acetohydroxyacid synthase
AHB: 2-Acetohydroxy-2-butyrate
AHV: 2-Acetohydroxy-2-valerate
AL: 2-Acetolactate
BCAA: Branched-chain amino acid
CSU: Catalytic subunit
ECF: Ethyl chloroformate
EHOP: 2-Ethyl-2-hydroxy-3-oxopentanoate
FID: Flame ionization detection
GC: Gas chromatography
MCF: Methyl chloroformate
MS: Mass spectrometry
PL: 2-Propionyllactate
PYR: Pyruvate
RI: Retention index
RSU: Regulatory subunit
TIC: Total ion chromatogram
