# Supplementary material for: An easy and sensitive assay for acetohydroxyacid synthases based on the simultaneous detection of substrates and products in a single step
Source: Anal Bioanal Chem. 2024 Oct 23;416(29):7085–98. doi: 10.1007/s00216-024-05613-1 (PMC11579085; doi:10.1007/s00216-024-05613-1)
Supplement: Supplementary file 1 — Supplementary file1 (PDF 1.12 MB) [file 216_2024_5613_MOESM1_ESM.pdf]

Supplementary information

Analytical and Bioanalytical Chemistry

An easy and sensitive assay for acetohydroxyacid synthases based on the simultaneous detection of substrates and products in a single step

Annika Engelhardt[a,b], Marco Ebeling[a,b], Elisabeth Kaltenegger[a], Dorothee Langel[a], and Dietrich Ober[a]

[a] Botanical Institute and Botanic Gardens, Kiel University, D-24098 Kiel, Germany

[b] Shared first authorship.

Contact: Dietrich Ober, Botanical Institute and Botanic Gardens, Kiel University, D-24098 Kiel, Germany, dober@bot.uni-kiel.de

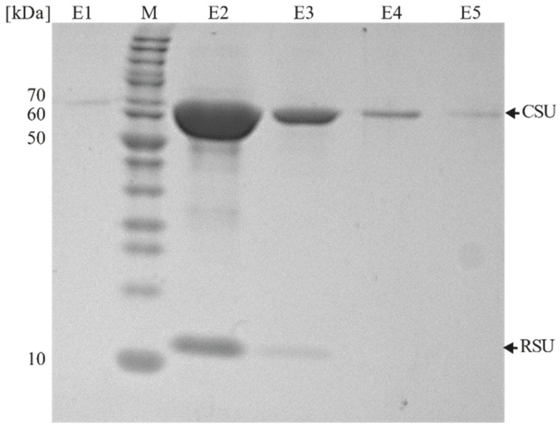

Fig. S1 SDS-PAGE of elution fractions (E1-E5) resulting from affinity purification of *Eco*AHASII. (12% gel, coomassie blue stained). The 6xHis-tagged catalytic subunit with a size of appx.64 kDa and the co-purified non-tagged RSU as described by Hill et al. [32]. M = protein size marker PageRuler™10-200 kDa (Thermo Fisher Scientific).

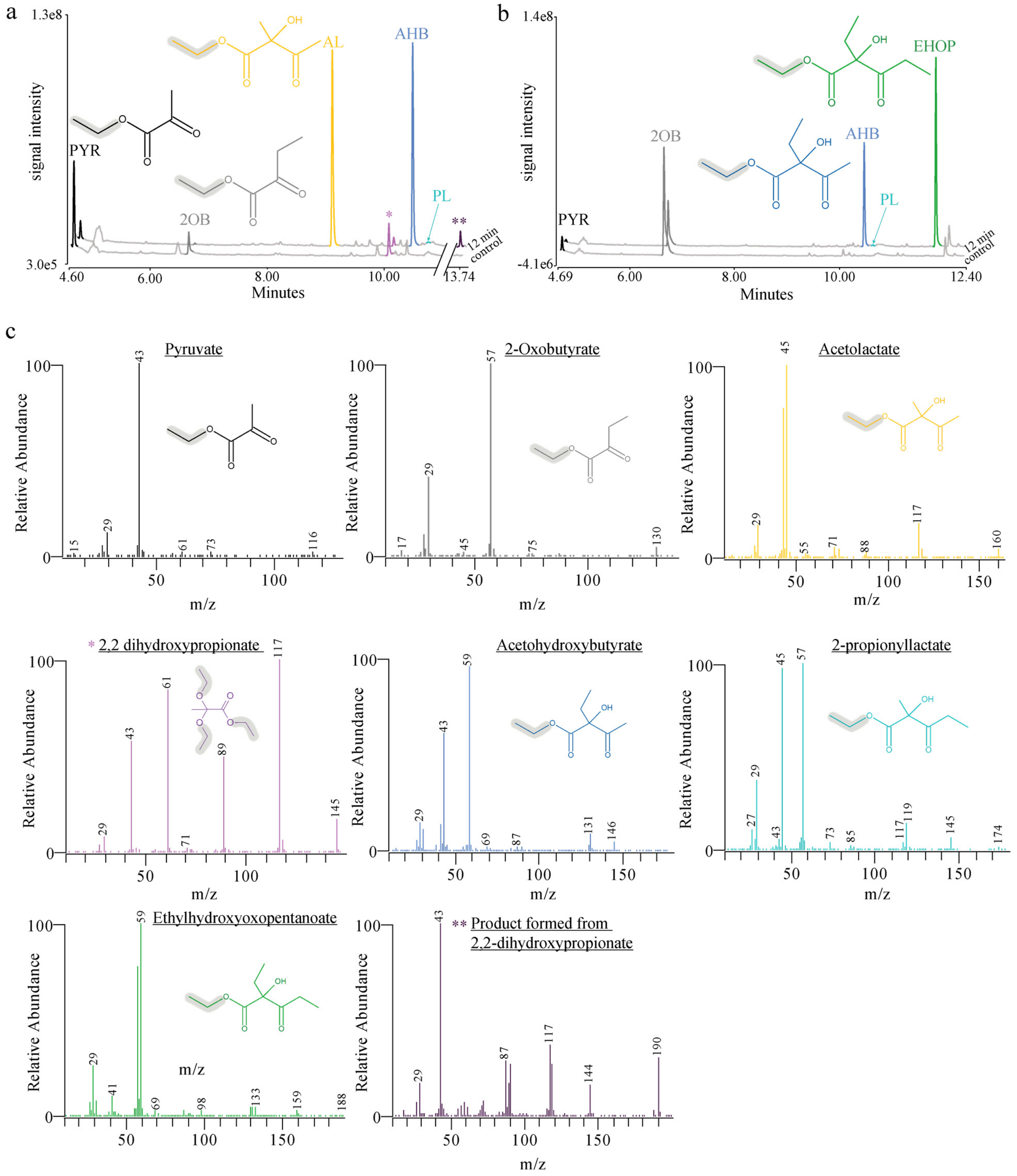

Fig. S2 Representative total ion chromatograms for the analysis of ECF-derivatized keto and acetohydroxyacids. Reactions with two different ratios of substrates were set up, one with 5 mM pyruvate (PYR) and 1 mM 2-oxobutyrate (2OB) (**a**) and one with 1 mM PYR and 5 mM 2OB (**b**). Chromatograms show a standard reaction assay without enzyme (control) and after incubation for 12 min at 30°C with 10 µg of affinity-purified *Eco*AHASII. Detectable products are 2-acetolactate (AL), 2-acetohydroxy-2-butyrate (AHB), 2-ethyl-2-hydroxy-3-oxopentanoate (EHOP), and propionyllactate (PL). The asterisk in **a** indicates traces of 2,2-dihydroxypropionate as a contaminant of PYR, which was shown to be converted by *Eco*AHAS II to a product of unknown structure **. **c** Respective Mass spectra to identified metabolites in chromatograms in **a** and **b**.

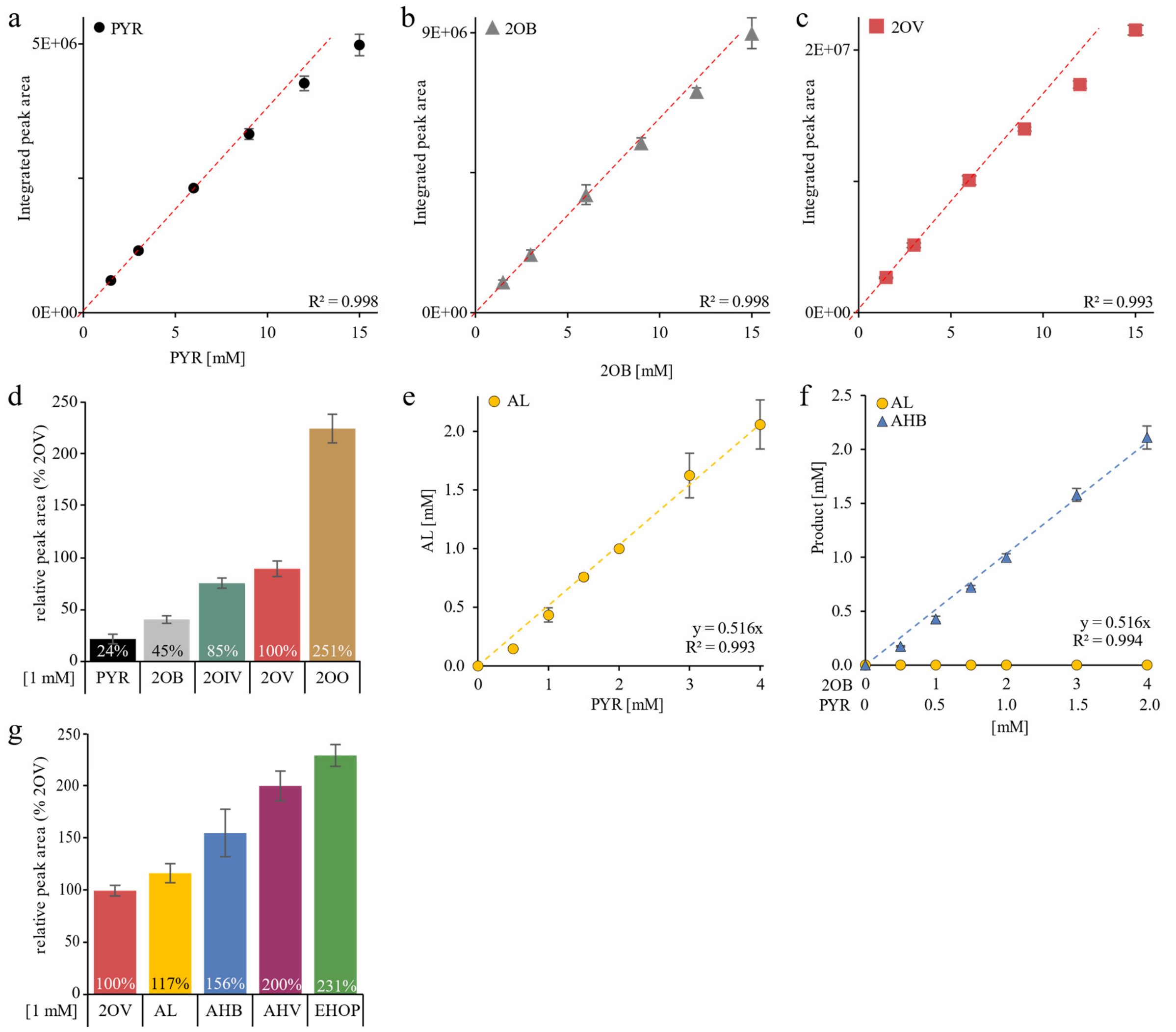

Fig. S3 MS detection of assay components, ethylated with ECF. Correlation between the integrated peak areas of the ECF derivatives of the substrates and the substrate concentration used for derivatization [1-15 mM] for pyruvate (PYR, **a**), 2-oxobutyrate (2OB, **b**), and 2-oxovalerate (2OV, **c**). The red dashed trend line crosses the origin and represents only the values for substrate concentrations from 0 to 9 mM. **d** MS response factors of the different ketoacids. PYR, 2OB, 2-oxoisovalerate (2OIV), 2OV, and 2-oxooctonate (2OO) were mixed (1 mM each) and derivatized with ECF. The integrated peak areas were related to that of 2OV (set at 100%). **e** Correlation between the amount of PYR as the sole substrate and the peak area of the product 2-acetolactate (AL). **f** Correlation between the concentrations of PYR and 2OB (in a ratio of 1:2) and the peak area of the product 2-acetohydroxy-2-butyrate (AHB). **g** MS response factors of the products (1 mM each) AL, AHB, 2-acetohydroxy-2-valerate (AHV), and 2-ethyl-2-hydroxy-3-oxopentanoate (EHOP) obtained from the values of the complete substrate conversions shown in E and F and from the incubation of 1 mM PYR in the presence of 5 mM 2OV resulting in the formation of AHB and the incubation of 2 mM and 4 mM 2OB as sole substrate for the formation of EHOP. 2OV was used as a standard and was set to 100%. Each value represents the mean ± SE of triplicate determinations.

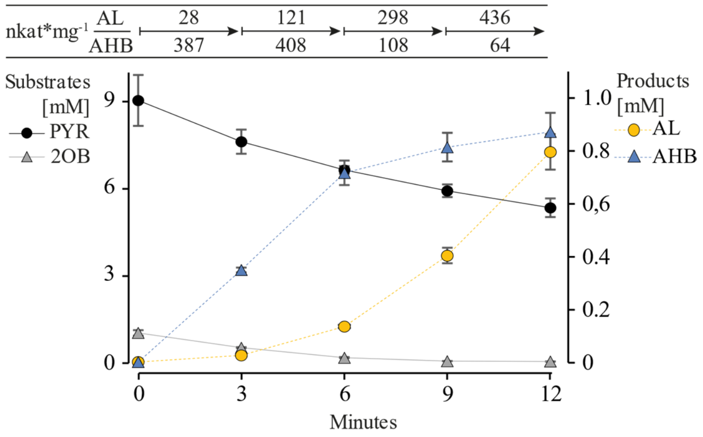

Fig. S4 MS detection of competition assay with pyruvate (PYR) and 2-oxobutyrate (2OB) as substrates. Time course of substrate and product concentrations in a reaction with 9 mM PYR and 1 mM 2OB and 5 µg/ml affinity-purified *Eco*AHASII. A 200 µl aliquot was taken every 3 minutes, the reaction was stopped, and substrates and products were quantified. Substrate scale from 0 to 9 mM, product scale from 0 to 1 mM. Each point represents the mean ± SE of triplicate determinations.

Table S1 Keto acid and acetohydroxyacid derivatives of the AHAS assay reactions ethylated with ECF. Retention indices (RI) on TG5-SILMS capillary column.

Substance	Formula	$[M]^+$	RI	MS data: characteristic ions m/z (% relative abundance)
Ketoacids				
Pyruvate ethyl ester (PYR)	$C_5H_8O_3$	116	848	14(1), 15(1), 25(1), 26(1), 27(5), 28(1), 29(13), 30(1), 41(1), 42(5), 43(100), 44(3), 45(4), 56(1), 57(1), 59(1), 61(2), 73(1), 91(2), 116(2)
2,2-dihydroxypropionate ethyl ester	$C_9H_{18}O_4$	190	1094	27(2), 29(5), 42(1), 43(23), 44(1), 45(1), 47(1), 61(33), 62(1), 88(1), 89(32), 90(2), 91(1), 117(100), 118(6), 119(1), 129(1), 145(21), 146(2), 147(1)
2-oxobutyrate ethyl ester (2OB)	$C_6H_{10}O_3$	130	926	15(1), 26(1), 27(9), 28(3), 29(35), 30(1), 41(1), 42(1), 55(1), 56(5), 57(100), 58(4), 59(1), 73(1), 74(1), 75(1), 87(1), 130(3)
2-oxovalerate ethyl ester (2OV)	$C_5H_{12}O_3$	144	995	26(1), 27(11), 28(2), 29(11), 38(1), 39(6), 40(1), 41(23), 42(8), 43(74), 44(3), 45(1), 55(4), 69(2), 70(6), 71(100), 72(5), 73(1), 88(2), 144(6)
2-oxoisovalerate ethyl ester (2OIV)	$C_5H_{12}O_3$	144	959	27(9), 28(1), 29(6), 39(7), 40(2), 41(28), 42(7), 43(100), 44(3), 55(2), 56(1), 57(1), 69(1), 70(14), 71(85), 72(4), 73(1), 88(1), 89(1), 144(2)
3-methyl-2-oxopentonate ethyl ester (3MOP)	$C_8H_{14}O_3$	158	1030	27(5), 28(2), 29(16), 39(5), 40(1), 41(28), 42(2), 43(1), 45(1), 53(1), 55(5), 56(7), 57(100), 58(5), 69(2), 84(6), 85(47), 86(3), 102(2), 158(1)
4-methyl-2-oxopentonate ethyl ester (4MOP)	$C_8H_{14}O_3$	158	1033	27(6), 29(16), 39(9), 40(2), 41(35), 42(9), 43(14), 45(2), 55(3), 56(2), 57(100), 58(4), 69(6), 83(3), 84(2), 85(95), 86(5), 88(3), 115(2), 158(4)
2-oxooctonate ethyl ester (2OO)	$C_{10}H_{18}O_3$	186	1358	27(6), 28(2), 29(11), 39(5), 41(18), 42(6), 43(70), 44(3), 55(12), 56(4), 57(23), 68(2), 69(4), 83(2), 84(2), 85(51), 86(3), 95(7), 113(100), 114(8), 186(1)
Acetohydroxyacids				
Acetolactate ethyl ester (AL)	$C_7H_{12}O_4$	160	1066	18(3), 27(6), 28(4), 29(15), 31(6), 41(3), 42(4), 43(78), 44(6), 45(100), 46(2), 55(3), 56(1), 71(4), 73(5), 75(2), 88(4), 117(17), 118(3), 160(4)
2-acetohydroxy-2-butyrate ethyl ester (AHB)	$C_8H_{14}O_4$	174	1118	27(5), 28(3), 29(14), 31(9), 39(3), 41(13), 42(4), 43(62), 44(3), 45(3), 55(2), 57(3), 58(4), 59(100), 60(3), 69(3), 87(2), 130(3), 131(7), 146(4)
2-propionyllactate ethyl ester (PL)	$C_8H_{14}O_4$	174	1127	26(2), 27(11), 28(6), 29(37), 30(1), 41(2), 43(5), 44(2), 45(98), 46(2), 55(4), 56(6), 57(100), 58(5), 73(4), 85(2), 117(4), 118(1), 119(14), 145(6)
2-acetohydroxy-2-valerate ethyl ester (AHV)	$C_9H_{16}O_4$	188	1256	26(1), 27(10), 28(2), 29(12), 38(1), 39(6), 40(1), 41(23), 42(7), 43(72), 44(3), 45(1), 55(4), 69(2), 70(5), 71(100), 72(5), 73(1), 88(2), 144(5)
2-ethyl-2-hydroxy-3-pentanoate ethyl ester (EHOP)	$C_9H_{16}O_4$	188	1257	27(5), 28(3), 29(22), 31(9), 41(13), 55(3), 56(4), 57(73), 58(7), 59(100), 60(3), 69(3), 87(3), 90(3), 98(4), 130(7), 131(7), 133(5), 159(4), 160(3)
Product formed from 2,2-dihydroxypropionate	?	?	1377	27(7), 29(17), 43(100), 44(6), 45(4), 55(3), 57(6), 59(7), 61(5), 71(6), 72(8), 87(29), 88(4), 59(17), 90(27), 115(4), 117(37), 118(27), 144(16), 190(30)
2-acetohydroxy-2-octonate ethyl ester (AHOO)	$C_{12}H_{22}O_4$	230	1485	32(9), 41(5), 43(68), 55(63), 57(7), 67(4), 69(7), 74(7), 81(5), 83(5), 85(9), 91(5), 95(7), 96(6), 97(100), 98(11), 107(5), 115(19), 146(15), 147(4)
Branched-chain amino acids				
D/L Valine ethyl ester	$C_7H_{15}O_2N$	145	1387	28(1), 29(3), 44(2), 55(11), 56(4), 70(2), 72(14), 74(5), 83(1), 98(13), 100(4), 101(7), 102(9), 116(38), 117(2), 128(1), 129(4), 144(100), 145(8)
D/L Leucine ethyl ester	$C_7H_{15}O_2N$	159	1461	29(2), 30(4), 43(3), 44(5), 58(6), 72(5), 74(3), 86(5), 101(3), 102(43), 103(2), 112(2), 114(3), 115(2), 116(2), 129(3), 130(2), 142(3), 158(100), 159(9)

Table S2 Keto acid and acetohydroxyacid derivatives of the AHAS assay reactions methylated with MCF. Retention indices (RI) on TG5-SILMS capillary column.

**Substance**	**Formula**	**$[M]^+$**	**RI**	**MS data: characteristic ions m/z (% relative abundance)**
Keto acids				
Pyruvate methyl ester (PYR)	$C_4H_6O_3$	102	663	14(5), 15(25), 16(1), 17(1), 28(3), 29(5), 30(1), 41(2), 42(10), 43(100), 44(3), 45(6), 47(2), 59(1), 73(2), 82(4), 83(2), 85(4), 102(10), 103(2)
2,2-dihydroxypropionate methyl ester	$C_6H_{12}O_4$	148	966	14(1), 15(10), 17(1), 29(4), 31(2), 42(4), 43(59), 44(2), 45(2), 47(12), 57(7), 58(1), 59(2), 75(3), 85(11), 86(1), 89(100), 90(4), 117(23), 133(2)
2-oxobutyrate methyl ester (2OB)	$C_5H_8O_3$	116	809	26(2), 27(10), 28(4), 29(36), 30(1), 31(1), 39(1), 41(1), 42(1), 43(1), 45(2), 55(1), 56(5), 57(100), 58(4), 73(1), 74(1), 75(2), 87(1), 130(3)
2-oxovalerate methyl ester (2OV)	$C_6H_{10}O_3$	130	932	15(15), 26(2), 27(16), 28(3), 29(4), 38(2), 39(12), 40(3), 41(43), 42(13), 43(100), 44(4), 45(2), 55(2), 59(6), 69(1), 70(2), 71(49), 72(2), 130(5)
2-oxoisovalerate methyl ester (2OIV)	$C_6H_{10}O_3$	130	871	15(16), 18(4), 27(13), 28(4), 29(3), 39(11), 40(2), 41(39), 42(11), 43(100), 44(4), 59(6), 70(3), 71(25), 130(2)
3-methyl-2-oxopentonate methyl ester (3MOP)	$C_7H_{12}O_3$	144	997	15(22), 26(2), 27(16), 28(9), 29(47), 39(18), 40(3), 41(87), 42(5), 45(2), 55(7), 56(9), 57(100), 58(5), 59(8), 69(3), 84(4), 85(86), 86(4), 144(3)
4-methyl-2-oxopentonate methyl ester (4MOP)	$C_7H_{12}O_3$	144	998	15(20), 27(10), 28(2), 29(23), 39(19), 40(3), 41(68), 42(17), 43(30), 45(2), 55(3), 57(61), 58(3), 59(10), 69(5), 83(3), 85(100), 86(5), 102(2), 144(4)
2-oxooctonate methyl ester (2OO)	$C_9H_{16}O_3$	172	1145	15(9), 27(8), 28(3), 29(10), 39(7), 40(1), 41(27), 42(9), 43(100), 44(3), 55(7), 56(2), 57(10), 59(4), 69(2), 85(43), 86(3), 95(6), 113(80), 114(6)
Acetohydroxyacids				
Acetolactate methyl ester (AL)	$C_6H_{10}O_4$	146	985	14(2), 15(22), 26(1), 27(4), 28(2), 29(11), 31(10), 39(2), 41(5), 42(6), 43(100), 44(4), 45(18), 55(1), 59(30), 71(2), 87(2), 103(28), 104(5), 146(7)
2-acetohydroxy-2-oxobutyrate methyl ester (AHB)	$C_7H_{12}O_4$	160	1047	15(17), 27(4), 29(8), 31(3), 39(5), 41(27), 42(7), 43(100), 44(5), 45(42), 55(2), 57(3), 59(18), 69(2), 73(24), 84(1), 116(7), 117(22), 118(5), 132(7), 160(1)
2-ethyl-2-hydroxy-3-oxopentanoate methyl ester (EHOP)	$C_8H_{14}O_4$	174	1099	15(21), 27(18), 28(7), 29(46), 39(11), 41(47), 42(6), 43(15), 45(67), 55(7), 57(100), 58(5), 59(25), 69(5), 73(49), 98(8), 116(12), 117(29), 119(16), 146(5), 174(2)
2-acetohydroxy-2-oxovalerate methyl ester (AHV)	$C_8H_{14}O_4$	174	1099	15(16), 27(6), 28(2), 29(7), 33(2), 39(6), 41(9), 42(4), 43(89), 44(5), 45(100), 46(2); 55(16), 57(5), 59(13), 73(5), 83(3), 87(3), 131(15), 132(17)
2-acetohydroxy-2-oxooctoate methyl ester (AHOO)	$C_{11}H_{20}O_4$	216	1244	15(7), 17(4), 27(5), 29(9), 39(6), 41(21), 42(5), 43(93), 44(5), 45(32), 55(49), 56(4), 59(15), 69(11), 71(4), 77(5), 88(5), 97(100), 98(9), 132(19), 173(1)
